# Potential applications of magnetic particles to detect and treat Alzheimer's disease

**DOI:** 10.1186/1556-276X-9-538

**Published:** 2014-10-01

**Authors:** Maria Antònia Busquets, Raimon Sabaté, Joan Estelrich

**Affiliations:** 1 Departament de Fisicoquímica, Facultat de Farmàcia, Universitat de Barcelona, Avda. Joan XXIII s/n, 08028 Barcelona, Catalonia, Spain; 2 Institut de Nanociència i Nanotecnologia (IN2UB), Universitat de Barcelona, C/ Martí i Franquès 1, 08028 Barcelona, Catalonia, Spain

**Keywords:** Superparamagnetic iron oxide nanoparticles, Gadolinium, Magnetic resonance imaging, Amyloid, Bio-barcode assay

## Abstract

Nanotechnology is an exciting and promising scientific discipline. At the nanoscale, a material displays novel physical properties that offer many new and beneficial products and applications. In particular, magnetic nanoparticles - a core/shell nanoparticle - present considerable diagnostic and therapeutic potentials, and superparamagnetic iron oxide nanoparticles (SPIONs) are considered promising theranostic tools. Alzheimer's disease (AD) is a neurodegenerative disorder that predominantly affects people over 65 years of age. The disease is characterized by the presence of extracellular plaques in the brain which are formed by interwoven fibrils composed of variants of the β-amyloid peptide. Medication can temporarily retard worsening of symptoms, but only in the first stages of the disease; early detection is thus of crucial importance. This minireview covers the progress made in research on the use of magnetic nanoparticles for *ex vivo* and/or *in vivo* detection and diagnosis of AD by means of magnetic resonance imaging (MRI), or to label peptides and fibrils. Of particular importance is the use of these nanoparticles to detect AD biomarkers in biological fluids. A description is given of the bio-barcode amplification assay using functionalized magnetic particles, as well as the use of such nanoparticles as a system for inhibiting or delaying the assembly of peptide monomers into oligomers and fibrils. Lastly, a brief overview is given of possible future lines of research in this.

## Review

### Introduction

Alzheimer's disease (AD) is a devastating neurodegenerative disorder that predominantly affects people over 65 years of age. The two main pathophysiological hallmarks of AD are the presence in the brain of extracellular amyloid plaques and of intracellular neurofibrillary tangles composed of hyperphosphorylated tau proteins. Although a causal relationship between amyloid deposits and the development of AD has not been conclusively demonstrated, a considerable body of experimental data suggests that amyloid aggregates are important in the etiology of AD [1-4]. The formation of such deposits is seen as the beginning of the neurodegenerative cascade that eventually leads to neurotoxicity, oxidative stress, and neuroinflammation. The amyloid core of these plaques consists of interwoven fibrils composed of β-amyloid (Aβ) peptide variants and surrounded by dead neurons. Aβ varies from 39 to 43 amino acids in length, the most abundant forms being 40 and 42 amino acids [5]. The initiating event of fibril formation is peptide misfolding, which leads to acquisition of the capacity to aggregate in an infinitely propagating fashion. Soluble spherical aggregates have been observed over the course of fibril formation and it is assumed that these spherical oligomers, usually called amyloid-β derived diffusible ligands (ADDLs), appear as intermediates in the pathway of fibril formation. Whereas early evidence suggested that Aβ fibrils initiate a cascade of events that result in neuronal cell death (*amyloid cascade hypothesis*) [6], a number of researchers have proposed that it is ADDLs, rather than monomers or insoluble amyloid fibrils, that may be responsible for synaptic dysfunction in AD (*revised amyloid hypothesis*) [7-11].

Although there is no cure, AD medications can temporarily slow the worsening of symptoms and improve the quality of life for those with Alzheimer's and their caregivers. Of the five US Food and Drug Administration (FDA) approved medications to treat the symptoms of AD, four are cholinesterase inhibitors (AChE) and the fifth is an *N*-methyl-d-aspartate (NMDA) receptor antagonist. The AChE inhibitors work by slowing down the disease activity that breaks down a key neurotransmitter. Donepezil, galantamine, rivastigmine, and tacrine are AChE inhibitors. Memantine is an NMDA that works by regulating the activity of glutamate, a chemical messenger involved in learning and memory. Memantine protects brain cells against an excess of glutamate, released in large amounts by cells damaged by AD and other neurological disorders.

The pharmacotherapy of AD offers primarily symptomatic benefits, and there is little or no evidence that it delays disease progression. Current research has therefore focused on identifying early detection and diagnosis strategies, one of the most challenging and crucial areas in modern medicine.

Nanotechnology is a multidisciplinary branch of science which encompasses numerous diverse fields of science and technology, ranging from biomedicine, pharmaceutics, agriculture, environmental sciences, advanced materials, chemical science, physics, electronics, information technology, and so on. Nanotechnology uses engineered materials or devices that have a functional organization on the nanometer scale in at least one dimension. The potential of nanotechnological applications to the life sciences arises from the fact that they exhibit bulk mesoscale and macroscale chemical and/or physical properties that are unique to the engineered material or device and not necessarily possessed by the molecules alone. The smaller size and high surface-to-volume ratio of nanoparticles are the key features which make them useful in biomedical fields, facilitating the development of many new properties, ease of functionalization, conjugation of biomolecules, etc. [12]. Nanodevices and nanomaterials aimed at biomedical applications are designed to interface and interact with biological systems at molecular levels with a high degree of specificity. Thus, they can stimulate, respond to, and interact with target cells and tissues in controlled ways to induce desired physiological responses while minimizing undesirable effects. With all this potential, nanotechnology could have a revolutionary impact on therapy, and in particular on AD diagnosis.

Nanoparticles used in biomedical applications include liposomes, polymeric micelles, block ionomer complexes, dendrimers, inorganic and polymeric particles, nanorods and quantum rods [13]. At present, core-shell-structured nanoparticles are used in preference to simple nanoparticles for several biomedical applications because of their additional advantages. Core-shell nanoparticles have a core made of a material coated with another material. The advantages of these nanoparticles over simple nanoparticles include less toxicity, greater dispersibility, bio- and cytocompatibility, better conjugation with other bioactive molecules, and increased thermal and chemical stability [14]. In this review, we present the promising perspectives that nanotechnology, and more specifically magnetic particles, offers in research on AD diagnosis.

### Nanotechnology: magnetic particles

Among the diversity of available core-shell nanoparticles, superparamagnetic nanoparticles coated with either inorganic materials (silica, gold) or organic materials (phospholipids, fatty acids, polysaccharides, peptides or other surfactants and polymers) are promising candidates for a wide range of biomedical applications. Ferrous or ferric oxide is the main constituent of magnetic particles; ferromagnetic iron oxides, maghemite, *γ*-Fe_2_O_3_, and magnetite, Fe_3_O_4_, are the most frequently used materials and are also the only magnetic materials that are FDA-approved for use in humans [15], although pure transition metals such as cobalt and nickel are also employed. The magnetic properties of superparamagnetic iron oxide nanoparticles, known as SPIONs, are based on their inducible magnetization, and they can be directed to a defined location in the presence of an externally applied AC magnetic field. This characteristic renders them attractive for many applications, ranging from various separation techniques and contrast-enhancing agents for magnetic resonance imaging (MRI) to drug delivery systems, as well as for magnetically assisted transfection of cells.

Magnetic nanoparticles are promising theranostic tools. They can be used in therapy (hyperthermia, local chemotherapy, and magnetically guided photodynamic therapy), drug delivery and diagnosis (for a review, see [16]). Due to the lack of an early diagnostic and therapeutic approach, theranosis is of considerable interest in AD, and SPIONs are considered particularly promising candidates due to their unique biocompatible and magnetic properties and their capacity for multifunctional application.

**Figure 1:**
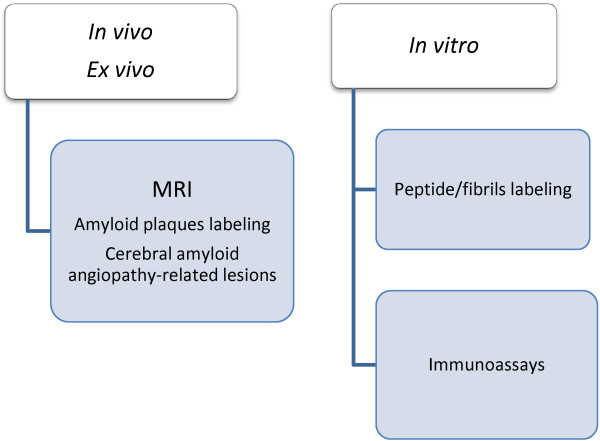
**Scheme of applications.** Scheme of the diagnostic applications of magnetic particles in AD.

Research on the use of magnetic nanoparticles in AD has focused on inhibiting β-peptide aggregation (according to the *amyloid cascade hypothesis*) and ADDL formation (if oligomers are thought to be responsible for initiating AD), or on developing sensitive methods to quantify biomarkers. It is in this latter field where the use of magnetic nanoparticles has had most impact in neurology. Figure 1 summarizes the different diagnostic applications of magnetic nanoparticles in AD.

### The blood–brain barrier: a challenge for nanotechnology

The main challenge in the diagnosis (and treatment) of AD is to overcome the restrictive mechanism of the blood–brain barrier (BBB). The BBB is the interface between the blood and the brain and consists of a diffusion barrier which impedes the influx of most compounds from blood to brain. It is composed of three cellular elements of the brain microvasculature: endothelial cells, astrocyte end-feet, and pericytes. BBB endothelial cells differ from endothelial cells in the rest of the body due to their absence of fenestrations, more extensive tight junctions, and sparse pinocytic vesicle transport [17]. Despite the extremely large surface area of the 100 billion capillaries contained in the human brain (approximately 20 m^2^), the permeability of many substances is low because of the dense anatomical structure of tight junctions between endothelial cells around the capillaries in the central nervous system (CNS). These tight junctions are composed of smaller subunits, frequently transmembrane proteins [18], each of which is anchored to the endothelial cells by another protein complex. This barrier does not exist in normal circulation, but in the brain it limits the flux of hydrophilic molecules across the BBB. In contrast, small lipophilic substances such as water and some gases (O_2_, CO_2_) diffuse freely across plasma membranes along their concentration gradient, and nutrients crucial to neural function such as glucose and amino acids enter the brain via transporters. However, the BBB restricts the passage of solutes and prevents the passage of most circulating cells and molecules, thus protecting the brain from foreign substances and maintaining CNS homeostasis [19]. Delivering therapeutic agents to the brain is a major challenge in the treatment of most brain disorders. For instance, one way of bypassing the BBB is the infusion of hyperosmolar solutions (e.g., of mannitol) in conjunction with the suitable drug. In consequence, one requirement for any substance capable of detecting biomarkers or inhibiting β peptide aggregation is that it should be able to cross the BBB.

One particularly important area in which nanotechnology is applied to the CNS is in the development of technologies and approaches for delivering drugs and other small molecules such as genes, oligonucleotides, and contrast agents across the BBB. An important advantage of a nanotechnological approach, as compared with the administration of a free drug, is that the crucial requirements needed to reach the CNS while producing minimal systemic effects can be met by supporting parts of the engineered complex.

### Magnetic particles for AD diagnosis

Early diagnosis in AD before the onset of marked symptoms is critical to prevent irreversible neuronal damage that eventually leads to dementia and ultimately death. The clinical development of positron emission tomography (PET) and single photon emission computed tomography (SPECT) imaging agents that target aggregated Aβ peptide deposits *in vivo* shows tremendous promise for detecting changes in the accumulation of senile plaques in living patients. Another important imaging technique which is potentially useful as a means to monitor the pathological progression of AD is MRI. Despite the relatively high quality of MR images of soft tissues, in some cases it is not possible to acquire sufficient image contrast to diagnose the pathology of interest. In such cases, contrast agents are generally used, which shorten the spin–lattice T_1_ and/or spin-spin T_2_ relaxation times of the water protons within the tissues/regions to which they are delivered, thus enhancing image contrast. Thus, what is imaged is not the contrast agent but rather its effect on the relaxivity of the adjacent water protons, predominantly through dipolar interaction [20]. Since the formation of senile plaques precedes neurofibrillary degeneration, most research has focused either on detection or identification of amyloid plaques, using SPIONs as negative contrast agents. These decrease MR signal intensity in the regions to which they are delivered, rendering these regions darker in the image. Paramagnetic substances such as gadolinium (Gd) are positive contrast agents, enhancing MR signal intensity. However, due to the toxicity associated with Gd-based contrast agents, the focus of research has shifted to SPIONs. Moreover, a more detailed analysis has shown that SPIONs also lead to a shortening of T_1_, resulting in a positive contrast [21]. To date, most formulations based on SPIONs have been approved as MRI contrast agents by the Food and Drug Administration as well as the European Medicines Agency; however, at the present, many of them are being taken off the market.

**Figure 2:**
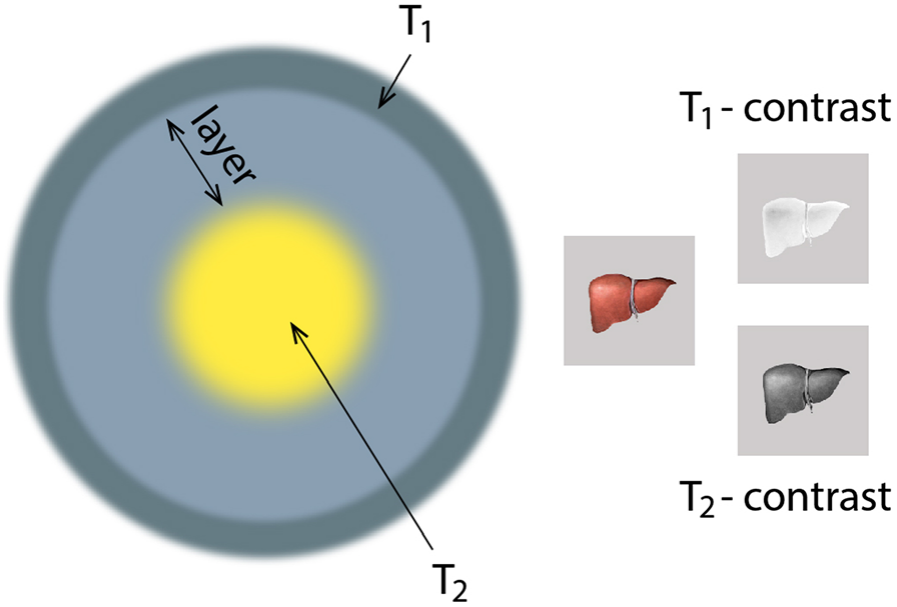
**Contrast agents used in MRI.** Possible structure of a nanoparticle containing both contrast agents. The thickness of the separation layer controls magnetic coupling between T_1_ and T_2_ contrast materials.

Conventional MRI contrast agents are generally only effective in a single imaging mode of either T_1_ or T_2_, and frequently present diagnostic ambiguities, especially for small biological targets. The combination of simultaneously strong T_1_ and T_2_ contrast effects in a single contrast agent could constitute a new breakthrough, since it has the potential to provide more accurate MR imaging. Due to strong magnetic coupling between T_1_ and T_2_ contrast agents when they are in close proximity, the spin–lattice relaxation processes of T_1_ contrast materials are significantly diminished. One of the strategies to overcome such a phenomenon is to locate the contrast agents in separate zones within the particle, for instance, one in the core and the other in the shell (Figure 2) [22].

In 1992, magnetite was found in the human brain [23] and studies since then have associated elevated levels of biogenic magnetite with both aging and AD [24-26]. As the magnetic susceptibility of magnetite is more than two orders of magnitude higher than that of ferrihydrite (the form in which most iron in the body is stored within the ferritin protein), the forces acting on biogenic magnetite particles in the body under the field and gradient conditions present in the 7-T whole body scanner and a 9.4-T small-bore research scanner have been studied with the aim of determining the threshold for effects on cellular ion channels. The results showed that the effect of these forces on the biogenic particles did not disrupt normal cellular function [27].

Magnetic nanoparticles are used as specific agents to detect amyloid plaques in AD. To this end, the particles must either be functionalized, for instance by incorporating antibodies against the Aβ on the particle's surface, or bound to the Aβ peptide. As Aβ has an inherent affinity with amyloid plaques, these targeted agents could selectively bind to amyloid plaques, thus enhancing the contrast from surrounding tissues and enabling plaque visualization by MRI. This approach was used in the study by Wadghiri et al. [28], in which magnetic nanoparticles covalently tethered to the *N*-terminus of Aβ(1–40) through amide coupling were synthesized for targeting and imaging of senile plaques in transgenic mice using MRI when co-injected with mannitol used to transiently open the BBB. In another *in vivo* study, Sillerud et al. [29] synthesized antibody-conjugated SPIONs targeted to the Aβ plaques in AD transgenic mice and were able to cross the BBB and detect the plaques in MR images; detectability was twofolds higher than in control cases.

**Figure 3:**
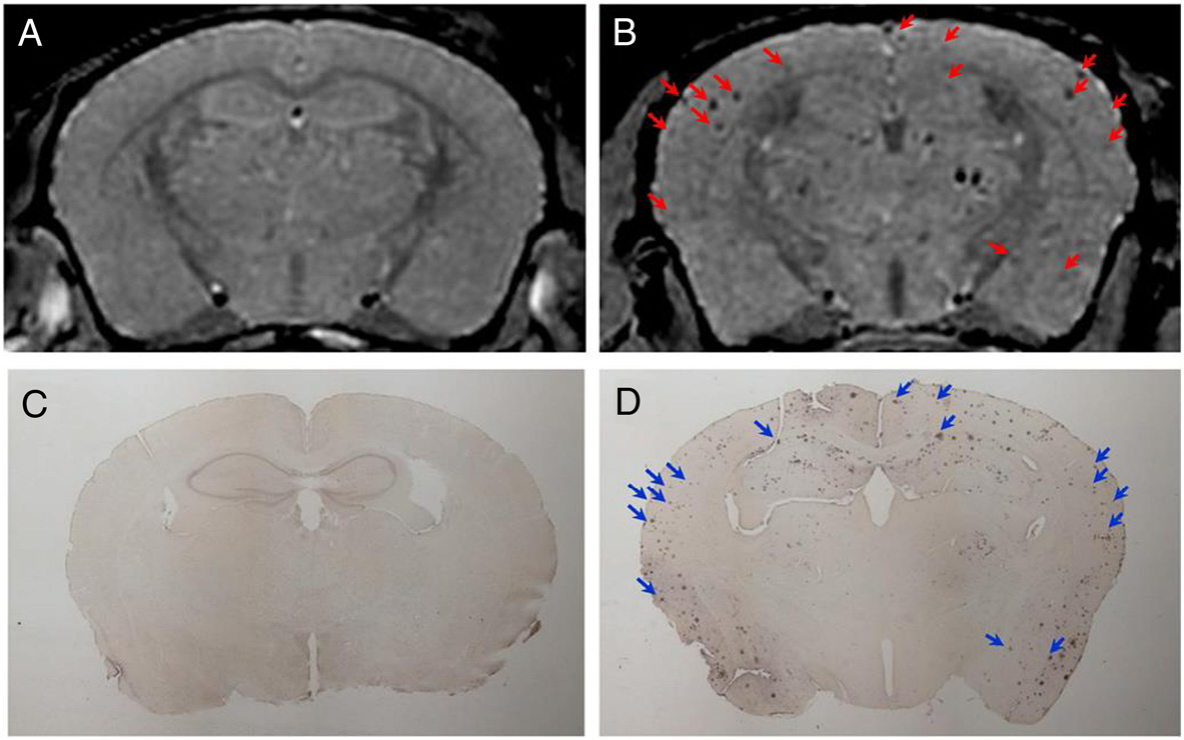
**Images of amyloid plaques.** Amyloid plaques detected with *in vivo* μMRI after intravenous femoral injection of SPIONs-Aβ(1–42) with mannitol. *In vivo* T_2_*-weighted MRI images show a 14-month-old wild-type **(A)** and transgenic **(B)** mouse brain. Note the match of many large plaques (arrowheads) in immunohistochemistry **(D)** and μMRI **(B)** in the AD transgenic mice. **(C)** Lack of plaque detection in a wild-type mouse following SPION-Aβ(1–42) injection (from [30], with permission).

As indicated, antibody-targeted magnetic nanoparticles have been used to detect Aβ plaques in AD. Following this approach, Yang et al. [30] used SPIONs chemically coupled with Aβ(1–42) to detect amyloid deposition together with mannitol for *in vivo* μMRI by femoral intravenous injection. The amyloid plaques detected were confirmed with matched histological sections. Results were compared between AD transgenic and wild mice for *in vivo* and *ex vivo* MRI brain imaging. Figure 3 compares the distribution of Aβ seen in histological sections with the matched patterns of hypointense spots.

Basing their study on the capacity of glycoconjugates (e.g., gangliosides and anionic glycol polymers) to bind Aβ [31], Kouyoumdjian et al. [32] have reported the preparation of glyconanoparticles. These particles were synthesized from dextran stabilized SPIONs. After the introduction of amine groups, sialic groups were linked to the SPIONs. The particles could selectively bind Aβ, and the assembly was detected by MRI both *in vitro* and *ex vivo* in mouse brains. Furthermore, the authors indicated that besides being nontoxic to neuroblastoma cells, the glyconanoparticles also reduced Aβ-induced cytotoxicity to cells.

Regarding T_1_ contrast agents, Poduslo et al. [33] visualized Aβ plaques with gadolinium diethylenetriaminepentaacetic acid (GdDTPA) conjugated to a putrescine-modified Aβ(1–40) capable of crossing the BBB and targeting amyloid deposits in the brain of transgenic mice. First, the Gd particles were incubated with brain sections. Results *in vitro* showed that putrescine modification enabled a dramatic increase in binding of Aβ to the plaques. Then, Gd particles were administrated intravenously to mice and Aβ plaques were labeled in brains of postmortem specimens. The authors of this study reported a more than ninefold enhancement (T_1_-weighted images) in cortex and hippocampus regions in AD transgenic mice at 7 T. Petiet et al. [34] have described a new method to detect amyloid plaques by means of *in vivo* MRI based on the intracerebroventricular injection of a non-targeted gadolinium-based contrast agent, which rapidly diffuses throughout the brain and increases the signal and contrast of MR images. This gain in image sensitivity after *in vitro* and *in vivo* gadolinium staining significantly improved the detection and resolution of individual amyloid plaques in the cortex and hippocampus of AD transgenic mice; furthermore, the improved image resolution was sufficiently sensitive to demonstrate an age-dependent increase in amyloid plaque load and a good correlation between the amyloid load measured by μMRI and histology. These results provided the first demonstration that non-targeted Gd staining could enhance the detection of amyloid plaques to monitor the progression of AD. Martins et al. [35] conjugated an optimized derivative of the Pittsburg compound-B (PiB), a well-established marker of Aβ plaques, to DO3A-monoamide, which is capable of forming stable, non-charged complexes with Gd (and other trivalent metal ions). Proton relaxivity evidenced binding of Gd (DO3A-PiB) to Aβ(1–40), resulting in a twofold increase in relaxivity as a consequence of immobilization of the complex. *Ex vivo* immunochemical studies showed that the DO3A-PiB complexes selectively targeted Aβ plaques in AD human brain tissue.

In other cases, bare magnetic particles have been utilized. Beckmann et al. [36] used SPIONs to noninvasively study microvascular lesions originated by cerebral amyloid angiopathy (CAA), a pathology exhibited by approximately 80% of the AD patients. CAA is characterized by deposition of Aβ on the walls of cerebral vessels, and is associated with hemorrhages ranging from microbleeds to stroke, and with vasculitis presenting a variable degree of lymphocyte infiltration and vascular thickening. Both conditions are accompanied by the presence of macrophages/microglia, which surround affected vessels. Detecting such events *in vivo* can help to better understand the role of CAA in the development of dementia. Foci of signal attenuation were detected by MRI in cortical and thalamic brain regions of aged transgenic mice. Attenuation is the consequence of the paramagnetic properties of the hemoglobin degradation product hemosiderin. Histology confirmed that foci of signal attenuation reflected an increase in CAA-related lesions. In this study, magnetic particles were injected intravenously to transgenic mice modeling AD. It was observed that 24 h after particle administration, when particles had been taken up by macrophages, the strength and number of foci of signal attenuation had increased. These results demonstrated that MRI in combination with the administration of SPIONs improves the detectability of CAA-related microvascular lesions in transgenic mouse models of AD.

In an *in vitro* study, Skaat and Mergel [37] combined magnetic and fluorescent imaging in one nanostructured system, using maghemite nanoparticles bearing either rhodamine or Congo red. The nanoparticles bound selectively to Aβ(1–40) fibrils in both cases. The Aβ(1–40)-nanoparticle assemblies thus formed were completely removed by a magnetic field. This hybrid system might enable the early detection of plaques and removal by magnetization.

Ferromagnetic metals such as Fe, Co, and Ni possess stronger magnetic moments than those of the currently widely used iron oxide nanoparticles; however, they cannot be used directly in biological applications due to their high reactivity in aqueous environments. To address this limitation, Choi et al*.*[38] described the synthesis of heterodimeric nanoparticles consisting of a cobalt magnetic core and a platinum shell directly fused to a gold nanoparticle (Co@Pt-Au). To obtain high colloidal stability and target specificity, lipoic acid-polyethylene glycol (PEG)-COOH complexes with disulfide ends that bind to the Au surface were ligated to the Co@Pt-Au nanoparticles. The terminal carboxyl groups of the PEG chains enabled covalent binding with lysine residues of neutravidin on the surface of the nanoparticles (Co@Pt-Au-neutravidin). These nanoassemblies presented high magnetization values. The MRI measurements of Co@Pt-Au-neutravidin nanoparticle samples mixed with an increasing amount of biotinylated Aβ(1–40) peptides showed that significant contrast changes took place when varying the peptide concentration. The results clearly showed that these nanoparticles can be used in MRI to monitor key structural stages of Aβ self-assembly. In particular, a significant change was observed in MRI signals during Aβ self-aggregation that corresponded to the detection of Aβ protofibrillar species in the early reversible stages of aggregation.

**Figure 4:**
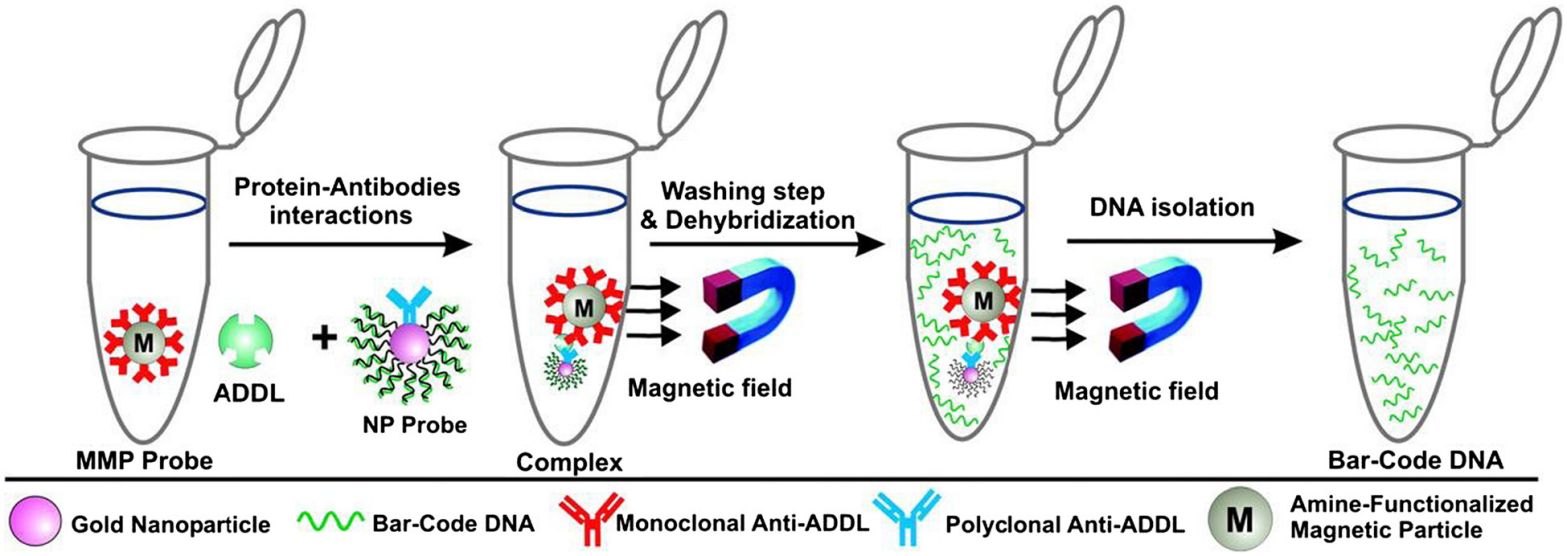
**The bio-barcode amplification assay.** The assay uses magnetic particles functionalized with monoclonal antibodies that recognize and bind ADDLs. The ADDLs are then sandwiched with a nanoparticle probe, modified with double-stranded DNA and an anti-ADDL polyclonal antibody. After repeated washing while using a magnet to immobilize the magnetic particles, a dehybridization step releases hundreds of barcode DNA strands for each antigen-binding event (From [39]. Copyright (2005) National Academy of Sciences, USA).

There is no definitive diagnosis of the disease other than *postmortem* identification of senile plaques and neurofibrillary tangles in the brain. *Premortem* diagnosis, based on a patient's clinical history, *in vivo* brain imaging, and neurophysiological, cognitive and neurological tests, is only 85% accurate. There are two general approaches for detecting soluble markers of AD. One approach is based on measuring total τ protein or amyloid β-protein concentrations in cerebrospinal fluid (CSF) or plasma. This approach is hampered by a significant overlap of such marker levels in healthy and unhealthy subjects and has led to inconclusive results. The other approach targets only the suspected pathogenic markers, such as cleaved τ protein, phosphorylated τ protein, or ADDLs. Although this approach to detecting pathogenic markers might lead to more definitive results, the concentrations of such markers in CSF are so low in the early stages of the disease that they cannot be identified accurately with conventional ELISA or blotting assays [39]. This limitation can be addressed by using the bio-barcode assay, an emerging diagnostic tool based on advances in nanotechnology and used for ultrasensitive enzyme-free detection of various protein and nucleic acid targets. In the case of proteins, the bio-barcode assay can be between one and six orders of magnitude more sensitive than conventional ELISA-based assays, depending on target and sample complexity [40,41]. Georganopoulou et al. [39] have demonstrated that the bio-barcode assay (Figure 4) can be used to determine approximate ADDL concentrations in CSF. The key factor to the bio-barcode assay is the homogeneous isolation of specific antigens by means of a sandwich process involving oligonucleotide-modified Au nanoparticles (nanoparticles with bio-barcodes) and magnetic nanoparticles, both functionalized with antibodies specific to the antigen of interest. The increased sensitivity gained derives mainly from very effective antigen sequestration and the amplification process that occurs as a result of the large number of barcode DNA strands released for each antigen recognition and binding event. The results of this study showed that ADDL levels in control subjects were lower than in subjects diagnosed with AD, with ADDL concentration medians in the two groups of ≈ 200 aM and 1.7 fM, respectively.

A collection of CSF samples requires lumbar puncture, a relatively risky and uncomfortable process which is therefore unsuitable for large-scale screening or repeated sampling for long-term monitoring of disease progression. Consequently, biomarkers in types of body fluid other than CSF are necessary, and one of the most promising body fluids is blood. However, biomarker concentrations in blood are very low and detection requires ultrahigh-sensitivity assay technologies [42]. The superconducting quantum interference device (SQUID) immunogenetic reduction (IMR) assay makes it possible to measure plasma biomarkers for diagnosis of AD since the association between the functionalized magnetic nanoparticles and the target biomarkers involves a reduction in magnetic susceptibility [43]. Yang et al. [44] prepared SPIONs biofunctionalized with antibodies against Aβ(1–40) and Aβ(1–42). In combination with the IMR technology, they demonstrated that such particles had the capacity to label Aβs specifically. In a further study by this same group [42], magnetic nanoparticles were coated with antibodies against Aβ(1–40), Aβ(1–42), and τ protein. The results showed that Aβ(1–42) and τ protein concentrations in healthy controls were significantly lower than those in patients with AD. Using this technology, the low detection limit for amyloids and τ protein was found to be 1 to 10 pg/mL.

Dopamine is a neurotransmitter, a chemical released by nerve cells to send signals to other nerve cells, and has recently been considered as a possible biomarker of AD. Basal dopamine levels are involved in basic brain functions, and disturbed concentration levels of this neurochemical have been associated with pathological states such as AD. Hence, determination of dopamine concentration levels plays an important role not only in the treatment of the disease but also in the study of basic brain functions. Ranc et al. [45] have presented a method for rapid analysis of dopamine levels in artificial CSF and mouse striatum samples. This method is based on a nanocomposite composed of magnetite and silver nanoparticles, the surface of which is modified with iron nitriloacetic acid, a dopamine selective compound. The magnetic properties of this nanocomposite enable simple separation of targeted molecules from a complex matrix while the silver acts as a platform for surface-enhanced Raman scattering (SERS). Rapid and simple quantitative experiments show improved detection of dopamine levels in biological assays at low femtomolar concentrations. The comparative data obtained from clinical samples of mouse striatum show that magnetic SERS is a strong alternative to conventional methods (HPLC-MS).

### Magnetic particles in the treatment of AD

**Figure 5:**
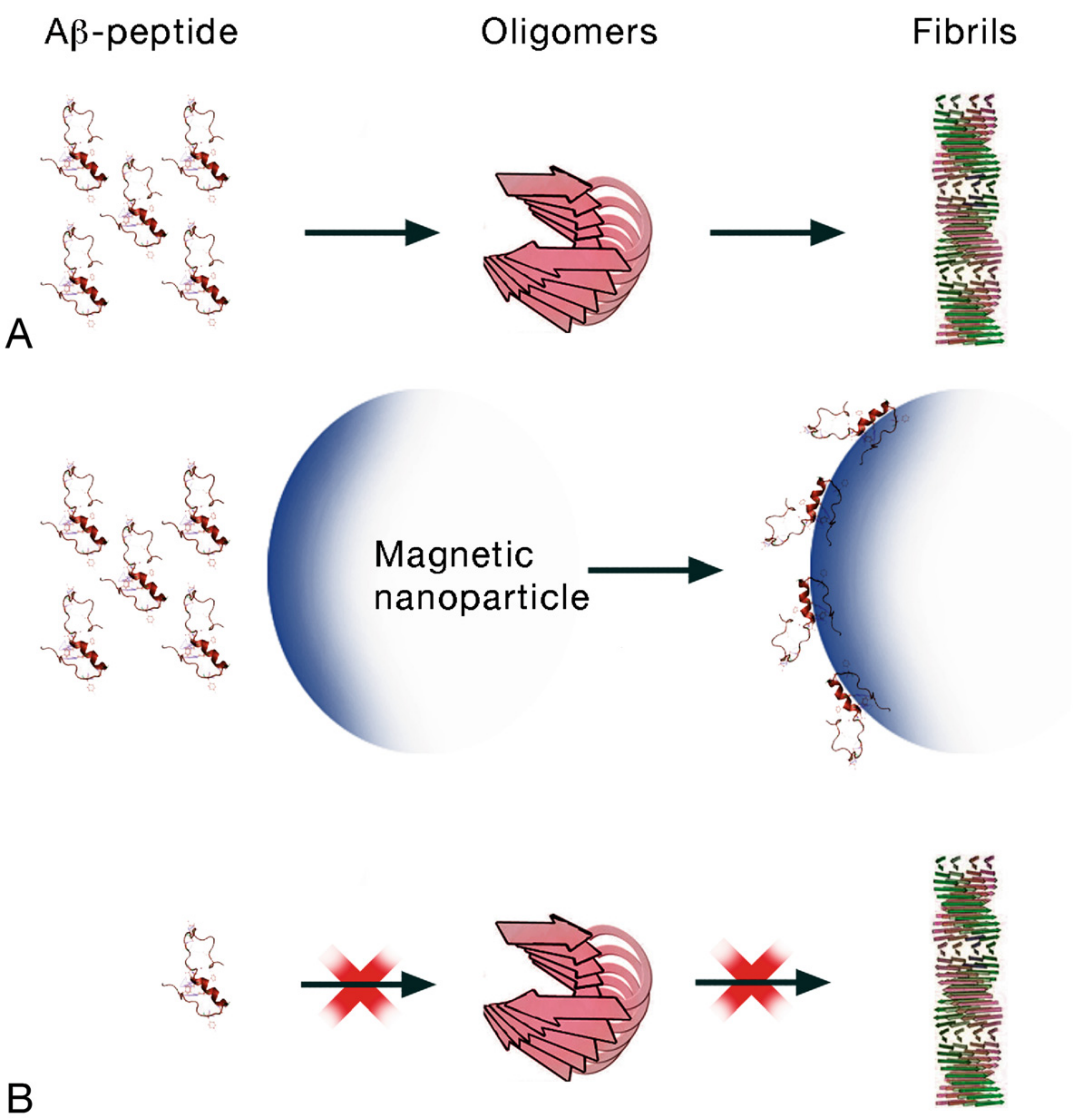
**Effect of magnetic particles on the fibrillogenesis of amyloid.** Schematic representation of the possible scenario of a reduction in the toxic effects of Aβ. **(A)** Usual sequence from peptide to fibrils. **(B)** Peptide monomers are adsorbed onto the surface of SPIONs and the free peptide concentration is thus lowered; consequently, the possibility of forming oligomers and fibrils is also reduced.

The pharmacotherapy of AD offers primarily symptomatic benefits, but does not retard disease progression. Consequently, researchers are seeking new ways to treat AD. There are several promising drugs in development, including use of enzymes to decrease Aβ production, substances that increase Aβ clearance, immunotherapy, enzymatic degradation of Aβ, inhibition of Aβ aggregation, antioxidants, anti-inflammatory drugs, statins, etc. (for a review, see [46,47]). From among these approaches, the inhibition of Aβ assembly has been considered the primary therapeutic strategy for this neurodegenerative disease. Any system considered for treating AD must possess the capacity to cross the BBB. To date, a large number of studies have described the effect of nanoparticulate systems on Aβ fibrillation [48]. Fibril formation occurs by means of nucleation-dependent kinetics where the formation of a critical nucleus is the key determining step, after which fibrillation proceeds rapidly. An exogenous material capable of reducing peptide toxicity may act by two opposite, postulated mechanisms: (1) by decreasing monomer nucleation and hence blocking aggregation, which would result in a reduction in the formation of ADDLs, fibrils, and plaques; or (2) by disaggregating amyloid plaques or fibrils. To date, it is the first mechanism (Figure 5) that has received most attention [49].

The results obtained from studying the kinetics of the process are inconclusive and extremely dependent on the experimental conditions of the *in vitro* studies. For instance, many years ago, it was demonstrated that cationic surfactants had a dual effect on Aβ fibrillation [50]; they could either accelerate or inhibit fibrillation depending on the surfactant concentration. A similar effect has been described for liposomes [51] and, more recently, for cationic polystyrene nanoparticles [52]. Depending on the specific ratio (i.e., between the peptide and particle concentration), the kinetic effects on amine-modified polystyrene nanoparticles vary between acceleration of the fibrillation process (at low particle surface area in solution) to inhibition of the fibrillation process at high surface area. However, the sole effect of negative surfactants is to delay the development of fibrils [53]. In contrast, Linse et al. [54] have reported that nanoparticles such as copolymer particles, cerium oxide particles, quantum dots, and carbon nanotubes, enhance the possibility of the appearance of a critical nucleus for fibril nucleation (referring to fibrils from human β-macroglobulin but extensible to any amyloid protein). Nanoparticles present high surface-to-volume ratios, and this enormous surface means that proteins may be bound onto the particle surface. The potentially high concentration of protein adsorbed at the particle surface, combined with the low dimensionality of the surface, can enhance the probability of partially unfolded proteins coming into frequent contact, promoting amyloid formation if the protein is suitable.

There are only a few reports on the interaction of SPIONs with amyloidogenic proteins in the literature, and the majority of these focus on insulin amyloid fibril formation. Only one of the reports [55] discusses the effect of magnetic nanoparticles on Aβ fibrillation, and more specifically, the effect of dextran polymer coating charge and thickness (i.e., a single polymer layer or a double polymer layer) on fibrillation kinetics. Depending on the surface coating charge, a dual surface area-dependent effect was observed, with lower concentrations of SPIONs inhibiting fibrillation, whereas higher concentrations enhanced the rate of Aβ fibrillation. The coating charge influenced the concentration at which acceleratory effects were observed, with positive SPIONs promoting fibrillation at significantly lower particle concentrations than either negatively charged or essentially uncharged SPIONs. The authors concluded that SPIONs designed for *in vivo* medical imaging applications should preferentially use a negatively charged or uncharged surface coating.

## Conclusions

Magnetic nanoparticles with paramagnetic or superparamagnetic properties are promising nanodiagnostic materials. Among the magnetic nanoparticles, SPIONs are excellent T_2_ contrast agents, although with an appropriately sized iron core and applied in moderate concentrations they also show a T_1_ contrast. Hence, due to their high biocompatibility, superparamagnetic properties, and their high capacity for use as multimodal contrast agents, these functionalized magnetic nanoparticles are considered the most promising magnetic nanoparticles in nanomedicine, and more specifically, in the diagnosis of AD. Moreover, as with other nanoparticulate systems, it has been suggested that SPIONs can modulate the fibrillation process either by inhibiting formation of the nucleus or by delaying transformation of ADDLs into fibrils. In this case, SPIONs could clearly be classified as theranostic agents. However, further studies on the interaction of SPIONs and Aβ are necessary to confirm the existence of such unique properties, especially *in vivo* research on animal models and/or *in vitro* research under more realistic conditions, for example in the presence of serum proteins, since it has been demonstrated that in a biological medium, the protein corona modifies and alters the properties of uncoated particles.

## Competing interests

The authors declare that they have no competing interests.

## Authors’ contributions

MAB, RS, and JE participated in the design of the review. JE drafted the manuscript. All authors read and approved the final manuscript.
